# Inhibition of Excessive Cell Proliferation by Calcilytics in Idiopathic Pulmonary Arterial Hypertension

**DOI:** 10.1371/journal.pone.0138384

**Published:** 2015-09-16

**Authors:** Aya Yamamura, Naoki Ohara, Kikuo Tsukamoto

**Affiliations:** Department of Pharmacy, College of Pharmacy, Kinjo Gakuin University, Nagoya, Japan; Georgia Regents University, UNITED STATES

## Abstract

Idiopathic pulmonary arterial hypertension (IPAH) is a rare and progressive disease of unknown pathogenesis. Vascular remodeling due to excessive proliferation of pulmonary arterial smooth muscle cells (PASMCs) is a critical pathogenic event that leads to early morbidity and mortality. The excessive cell proliferation is closely linked to the augmented Ca^2+^ signaling in PASMCs. More recently, we have shown by an siRNA knockdown method that the Ca^2+^-sensing receptor (CaSR) is upregulated in PASMCs from IPAH patients, involved in the enhanced Ca^2+^ response and subsequent excessive cell proliferation. In this study, we examined whether pharmacological blockade of CaSR attenuated the excessive proliferation of PASMCs from IPAH patients by MTT assay. The proliferation rate of PASMCs from IPAH patients was much higher (~1.5-fold) than that of PASMCs from normal subjects and patients with chronic thromboembolic pulmonary hypertension (CTEPH). Treatment with NPS2143, an antagonist of CaSR or calcilytic, clearly suppressed the cell proliferation in a concentration-dependent manner (IC_50_ = 2.64 μM) in IPAH-PASMCs, but not in normal and CTEPH PASMCs. Another calcilytic, Calhex 231, which is structurally unrelated to NPS2143, also concentration-dependently inhibited the excessive proliferation of IPAH-PASMCs (IC_50_ = 1.89 μM). In contrast, R568, an activator of CaSR or calcimimetic, significantly facilitated the proliferation of IPAH-PASMCs (EC_50_ = 0.33 μM). Similar results were obtained by BrdU incorporation assay. These results reveal that the excessive PASMC proliferation was modulated by pharmacological tools of CaSR, showing us that calcilytics are useful for a novel therapeutic approach for pulmonary arterial hypertension.

## Introduction

Pulmonary arterial hypertension (PAH) is caused by functional and structural changes in the pulmonary vasculature. Pulmonary vascular remodeling is triggered by a progressive elevation of pulmonary vascular resistance and pulmonary arterial pressure in patients with PAH. The elevated pulmonary arterial pressure induces extensive changes in heart structure followed by right heart failure, and eventually death. PAH is clinically defined by chronic increases of pulmonary arterial pressure due to various causes and resting mean pulmonary arterial pressure of ≥25 mmHg [1, 2]. The five-year survival rate of PAH after diagnosis is ~57%. In the United States, the mean age of PAH patients was 36.4 years in the 1980s, but it was 53.0 years in 2007, due to improved diagnosis, treatment, and management for PAH [3, 4].

Pulmonary vascular remodeling occurs due to the excessive proliferation of pulmonary arterial smooth muscle cells (PASMCs) [5, 6]. Cell proliferation is closely linked to Ca^2+^ mobilization and signaling in PASMCs. A major trigger for the PASMC proliferation is elevated cytosolic Ca^2+^ concentration ([Ca^2+^]_cyt_). In PASMCs, [Ca^2+^]_cyt_ is regulated by the balance of Ca^2+^ influx through Ca^2+^-permeable channels in the plasma membrane and Ca^2+^ release from the intracellular store sites. PASMCs express several Ca^2+^-permeable channels including voltage-dependent Ca^2+^ channels, receptor-operated Ca^2+^ channels, and store-operated Ca^2+^ channels [7–11]. It has been reported that receptor- and store-operated Ca^2+^ channels were upregulated in lung tissues and PASMCs from idiopathic pulmonary arterial hypertension (IPAH) patients, compared with PASMCs from normal subjects and normotensive patients, which resulted in enhanced Ca^2+^ signaling and excessive PASMC proliferation [12, 13]. These channels are also reported to be upregulated in PASMCs during hypoxia [14–18].

In addition to these Ca^2+^ influx pathways, more recently, we found that the extracellular Ca^2+^-sensing receptor (CaSR) was expressed at low levels in human PASMCs, and the expression level was upregulated in PASMCs from IPAH patients [19]. CaSR is classified as a member of the G-protein-coupled receptor subfamily C (also known as GPRC2A) [20, 21]. CaSR, which was originally identified from the parathyroid glands, senses the Ca^2+^ concentration in serum and regulates parathyroid hormone secretion to control serum Ca^2+^ concentration [22]. It has been reported that CaSR is also expressed in various mammalian tissues including kidney, bone, gastrointestinal tract, skin, brain, and the cardiovascular system [21, 23]. In addition, we previously demonstrated that the upregulation of CaSR enhanced the extracellular Ca^2+^-induced [Ca^2+^]_cyt_ increase in IPAH-PASMCs, contributing to enhanced Ca^2+^ signaling and excessive cell proliferation in IPAH-PASMCs [19].

Our previous report revealed that excessive cell proliferation due to enhanced CaSR function in IPAH-PASMCs was attenuated by the knockdown of CaSR with siRNA [19]. In this study, we examined whether pharmacological tools for CaSR modulated excessive cell proliferation in IPAH-PASMCs by MTT and BrdU incorporation assays. As pharmacological modulators for CaSR, a synthetic activator of CaSR (calcimimetic), R568, and antagonists of CaSR (calcilytics), NPS2143 and Calhex 231, were used in this investigation. Here, we found that the blockade of CaSR by calcilytics attenuated excessive cell proliferation in IPAH-PASMCs, but did not affect it in PASMCs from normal subjects and patients with chronic thromboembolic pulmonary hypertension (CTEPH).

## Materials and Methods

### Cell culture

Cell lines of PASMCs (passages 5 to 10) from normal subjects (Lonza, Walkersville, USA), IPAH patients [12], and CTEPH patients [24] were cultured in Medium 199 supplemented with 10% fetal bovine serum, 100 U/ml penicillin plus 100 μg/ml streptomycin (Invitrogen/GIBCO, Grand Island, USA), 50 μg/ml D-valine (Sigma-Aldrich, St. Louis, USA), and 20 μg/ml endothelial cell growth supplement (BD Biosciences, Franklin Lakes, USA) at 37°C.

### Cell proliferation assays

The PASMC proliferation was evaluated using Cell Counting Kit-8 (Dojin, Kumamoto, Japan) based on MTT (3-(4,5-dimethyl-2-thiazolyl)-2,5-diphenyl-2H-tetrazolium bromide) assay [25] and Cell Proliferation ELISA BrdU Colorimetric Kit (Roche Diagnostics, Mannheim, Germany) based on BrdU (bromodeoxyuridine) incorporation assay. PASMCs were subcultured in 96-well plates (approximately 1×10^4^ cells per well) and incubated at 37°C for 24~72 h. Cellular viability was quantified colorimetrically as the absorbance at 450 nm (A_450_) for MTT assay or 370 nm for BrdU incorporation assay using Benchmark Plus Microplate Reader and Microplate Manager (ver. 5.2; Bio-Rad Laboratories, Hercules, USA).

### Drugs

**Fig 1:**
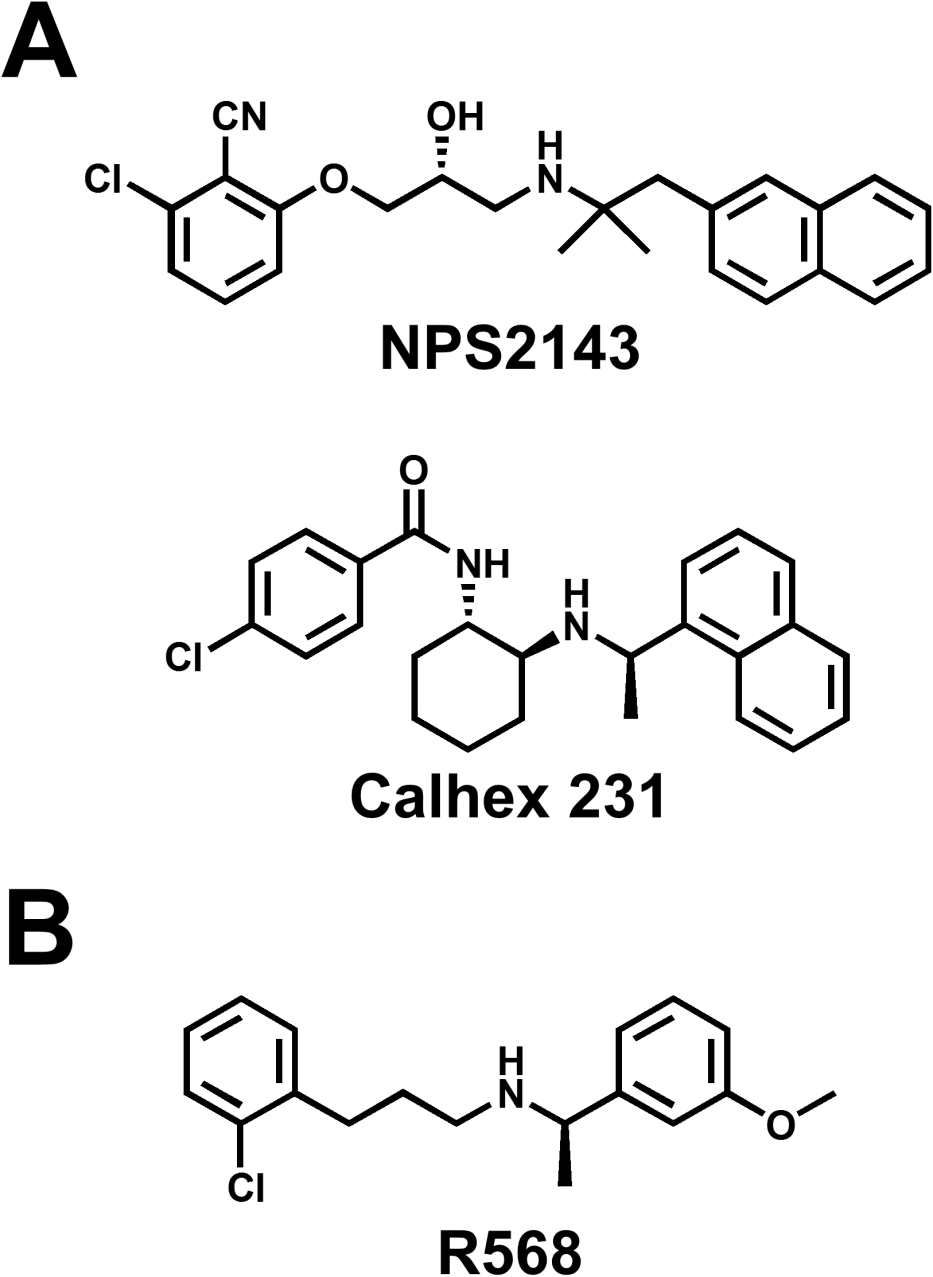
Chemical structure of CaSR antagonist (calcilytic) and agonist (calcimimetic). (**A**) The chemical structures of calcilytics, or negative allosteric modulators of CaSR, NPS2143 and Calhex 231. (**B**) The chemical structure of a calcimimetic, or a positive allosteric modulator of CaSR, R568.

The chemical structures of modulators for CaSR, NPS2143 (2-chloro-6-[(2R)-3-[[1,1-dimethyl-2-(2-naphthalenyl)ethyl]amino-2-hydroxypropoxy]benzonitrile) (Tocris Bioscience, Ellisville, USA), Calhex 231 (4-chloro-N-[(1S,2S)-2-[[(1R)-1-(1-naphthalenyl)ethyl]amino]cyclohexyl]benzamide) (Sigma-Aldrich), and R568 (2-chloro-N-[(1R)-1-(3-methoxyphenyl)ethyl]benzenepropanamine) (Tocris Bioscience) are shown in Fig 1. These hydrophobic compounds were dissolved in dimethyl sulfoxide at a concentration of 10 mM as a stock solution.

### Statistical analysis

Pooled data are shown as the mean±S.E. The statistical significance of differences between two groups was determined by Student’s t-test. The statistical significance of differences among groups was determined by Scheffé’s test after one-way analysis of variance (ANOVA). Significant differences are expressed in the figure as *p<0.05 or **p<0.01. The data of the relationship between drug concentrations and cell proliferation were fitted using the following equations: relative value (%) = 100-(100-C)/{1+(K_d_/[drug])^n^) (for inhibition) and A_max_/{1+(K_d_/[drug])^n^)+100 (for activation), where K_d_ is the apparent dissociation constant of drug (IC_50_ and EC_50_, respectively), [drug] is the concentration of drug, n is the Hill coefficient, C is the component resistant to drug, and A_max_ is the maximum value of the response.

## Results

### Excessive cell proliferation in PASMCs from IPAH patients

**Fig 2:**
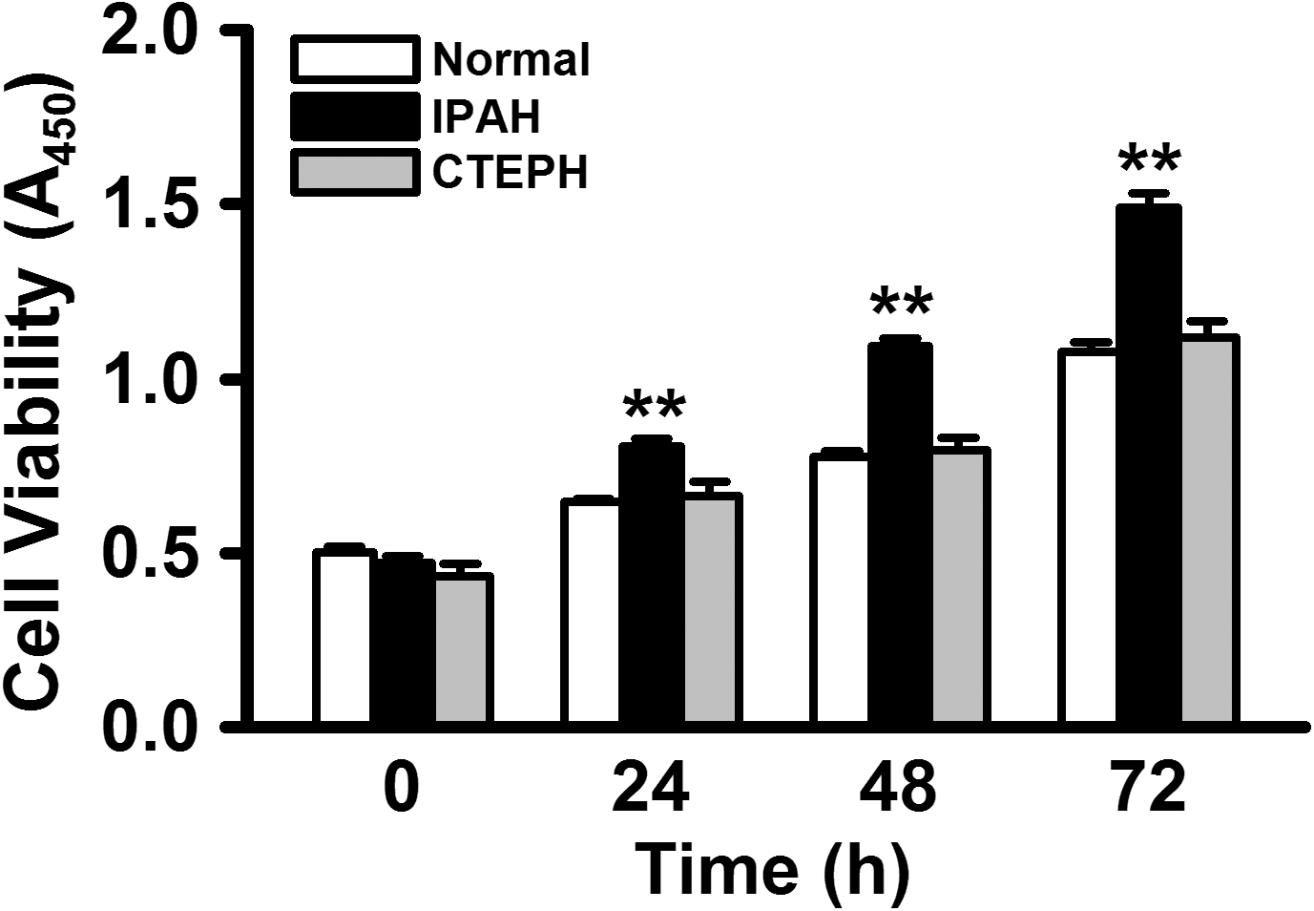
Excessive cell proliferation in PASMCs from IPAH patients. The proliferation rates of PASMCs from normal subjects, IPAH patients, and CTEPH patients were analyzed using quantitative colorimetric assay based on MTT test for cellular viability. When PASMCs were subcultured in 96-well plates, the cell number was made up to approximately 1×10^4^ cells per well. Summarized data show the proliferation rates of PASMCs from normal subjects, IPAH patients, and CTEPH patients. Data were obtained from 8 experiments. **p<0.01 vs. normal PASMCs.

At first, we analyzed the proliferation rates of PASMCs from normal subjects and patients with IPAH and CTEPH by quantitative colorimetric assay based on the MTT test for cellular viability. In normal PASMCs, the cell number was gradually increased until 72 h after subculture (A_450_ = 0.65±0.01 at 24 h, 0.78±0.01 at 48 h, and 1.08±0.03 at 72 h, n = 8 for each, p<0.01 vs. 0.50±0.02 at 0 h, n = 8; Fig 2). The proliferation rate in IPAH-PASMCs was much higher than that in normal PASMCs (0.81±0.02 at 24 h, 1.09±0.02 at 48 h, and 1.49±0.04 at 72 h, n = 8 for each, p<0.01 vs. 0.47±0.02 at 0 h, n = 8, and p<0.01 vs. normal PASMCs). On the other hand, the proliferation rate in CTEPH-PASMCs was similar to that in normal PASMCs (0.66±0.04 at 24 h, 0.79±0.04 at 48 h, and 1.12±0.05 at 72 h, n = 8 for each, p<0.01 vs. 0.44±0.03 at 0 h, n = 8, and p>0.05 vs. normal PASMCs). This result using a colorimetric cell counting kit was mostly consistent with our previous data using an automated cell counter [19].

### Inhibitory effect of NPS2143 on excessive proliferation of PASMCs from IPAH patients

**Fig 3:**
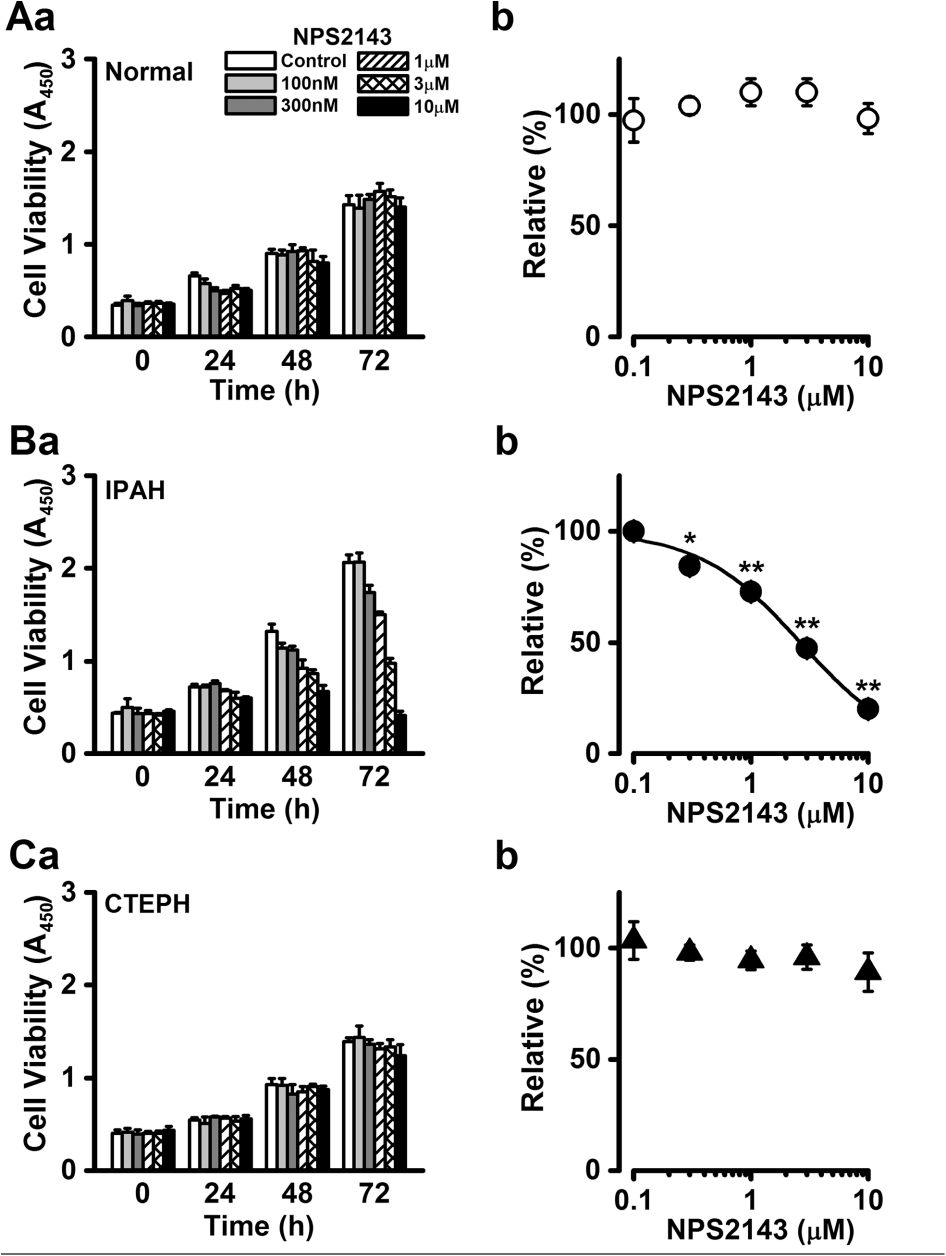
Inhibitory effect of NPS2143 on enhanced proliferation of PASMCs from IPAH patients. The effects of NPS2143, which is widely used as a calcilytic or negative allosteric regulator of CaSR, on cell proliferation were examined in normal, IPAH, and CTEPH PASMCs. Summarized data (**a**) show the effect of NPS2143 on cell viability at the concentration range between 100 nM and 10 μM in normal (**A**), IPAH (**B**), and CTEPH (**C**) PASMCs. Dose-response curve (**b**) of cell proliferation at 72 h for NPS2143 in normal (**A**), IPAH (**B**), and CTEPH (**C**) PASMCs. The absorbance at several concentrations of NPS2143 was normalized by the value in the absence of NPS2143 (as 100%). The IC_50_ value of NPS2143 for the proliferation of IPAH-PASMCs was 2.64 μM and the Hill coefficient was 1.00. Data were obtained from 5~13 experiments. *p<0.05 or **p<0.01 vs. control (100%).

NPS2143 is used as a calcilytic, or negative allosteric modulator of CaSR, and therefore blocks CaSR function. The cell survival of normal PASMCs was not affected by the treatment with NPS2143 regardless of culture time (98±7% at 10 μM and 72 h, n = 5, p>0.05 vs. control (100%), n = 5; Fig 3A). In contrast, the proliferation rate of IPAH-PASMCs was significantly reduced in the presence of 10 μM NPS2143 after 48 h (49% decrease, n = 3, p<0.01 vs. control) and 72 h (80% decrease, n = 10, p<0.01 vs. control) culture (Fig 3Ba). In the next set of experiments, the concentration dependence of the growth inhibition by NPS2143 was analyzed in IPAH-PASMCs. As the inhibitory effect by NPS2143 at 72 h culture was most prominent (Fig 3Ba), we evaluated the dose-response relationship of PASMC proliferation upon 72 h culture. The NPS2143-induced growth inhibition occurred in a concentration-dependent manner (20±2% at 10 μM, n = 10, p<0.01; Fig 3Bb). The IC_50_ value of NPS2143 was 2.64 μM, and the Hill coefficient was 1.00 (n = 7~13). On the other hand, the proliferation of CTEPH-PASMCs was not influenced by the presence of NPS2143 (89±9% at 10 μM and 72 h, n = 5, p>0.05 vs. control; Fig 3C).

### Attenuation of excessive cell proliferation by Calhex 231 in PASMCs from IPAH patients

**Fig 4:**
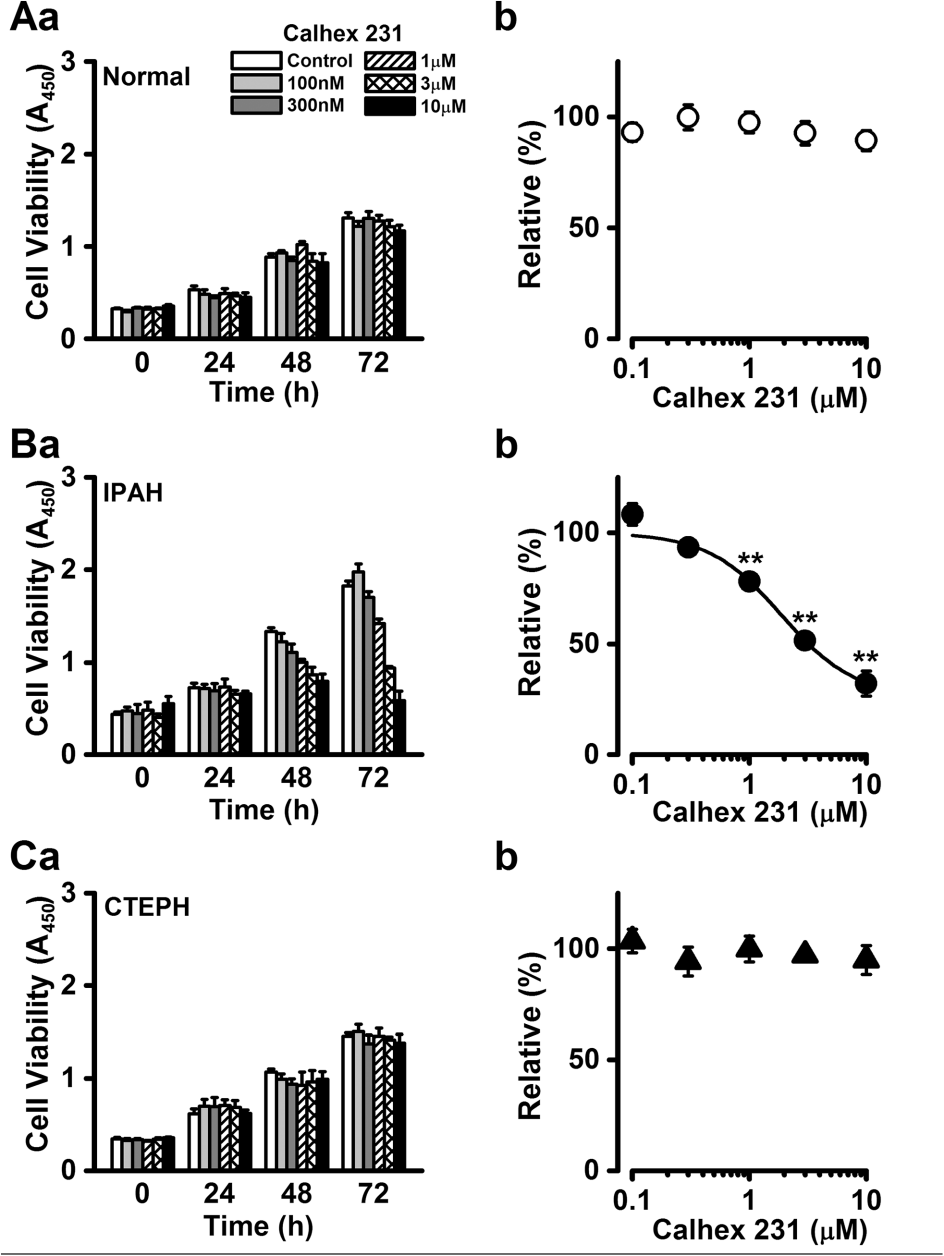
Attenuation of cell proliferation by Calhex 231 in PASMCs from IPAH patients. The effects of Calhex 231, a calcilytic structurally-unrelated to NPS2143, on cell proliferation were examined in normal, IPAH, and CTEPH PASMCs. Summarized data (**a**) show the effect of Calhex 231 on the cell viability at the concentration range between 100 nM and 10 μM in normal (**A**), IPAH (**B**), and CTEPH (**C**) PASMCs. Dose-response curve (**b**) of cell proliferation at 72 h for Calhex 231 in normal (**A**), IPAH (**B**), and CTEPH (**C**) PASMCs. The absorbance at several concentrations of Calhex 231 was normalized by the value in the absence of Calhex 231 (as 100%). The IC_50_ value of Calhex 231 for the proliferation of IPAH-PASMCs was 1.89 μM and the Hill coefficient was 1.39. Data were obtained from 5~8 experiments. **p<0.01 vs. control (100%).

To confirm that the inhibitory effect of NPS2143 on excessive cell proliferation was mediated by CaSR antagonism in IPAH-PASMCs, the effect of Calhex 231, which is another calcilytic structurally unrelated to NPS2143, on cell proliferation was examined in normal, IPAH, and CTEPH PASMCs. The cell survival of normal PASMCs was not affected by the presence of Calhex 231 (89±4% at 10 μM and 72 h, n = 8, p>0.05 vs. control; Fig 4A). When IPAH-PASMCs were incubated with medium containing various concentrations of Calhex 231 for 72 h, Calhex 231 dramatically inhibited cell growth in a concentration-dependent manner (32±6% at 10 μM, n = 6, p<0.01 vs. control; Fig 4B) with an IC_50_ value of 1.89 μM and a Hill coefficient of 1.39 (n = 6). In CTEPH-PASMCs, there was no significant difference in cell proliferation in the presence of Calhex 213 (95±6% at 10 μM, n = 5, p>0.05 vs. control; Fig 4C).

### Enhancement of cell proliferation by calcimimetic in PASMCs from IPAH patients

**Fig 5:**
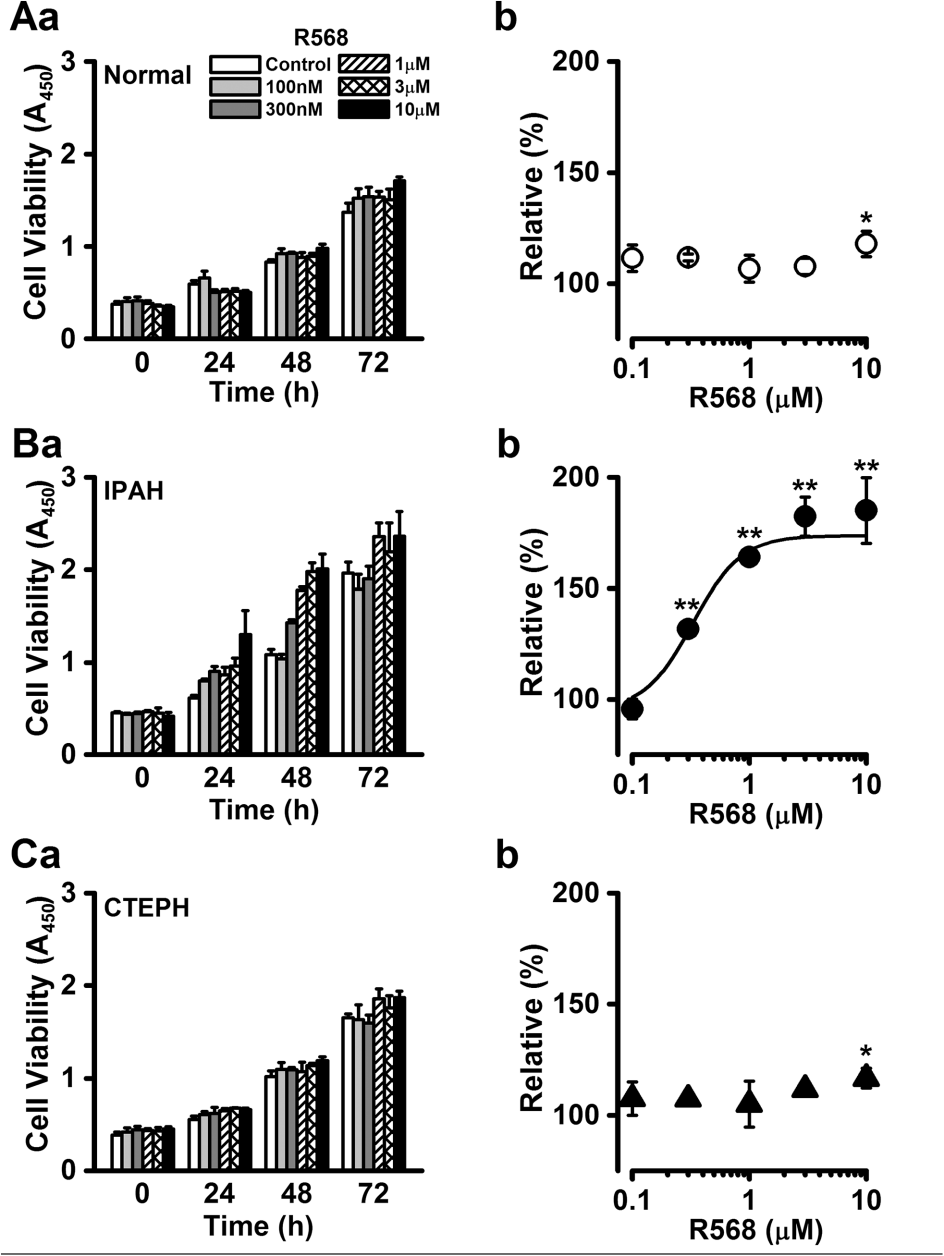
Enhancement of cell proliferation by a calcimimetic in PASMCs from IPAH patients. The effects of R568, a calcimimetic or positive allosteric regulator of CaSR, on cell proliferation were examined in normal, IPAH, and CTEPH PASMCs. Summarized data (**a**) show the effect of R568 on the cell viability in the concentration range between 100 nM and 10 μM in normal (**A**), IPAH (**B**), and CTEPH (**C**) PASMCs. Dose-response curve (**b**) of cell proliferation at 48 h for R568 in normal (**A**), IPAH (**B**), and CTEPH (**C**) PASMCs. The absorbance at several concentrations of R568 was normalized by the value in the absence of R568 (as 100%). The EC_50_ value of R568 for the proliferation of IPAH-PASMCs was 0.33 μM and the Hill coefficient was 2.10. Data were obtained from 5~11 experiments. *p<0.05 or **p<0.01 vs. control (100%).

Alternatively, the effect of the calcimimetic R568, a synthetic activator and positive allosteric modulator of CaSR, on cell proliferation was examined in normal, IPAH, and CTEPH PASMCs. The proliferation rate of normal PASMCs was slightly increased by the treatment with R568 at the highest concentration of 10 μM after 48 h culture (118±6% at 48 h, n = 6, p = 0.022 vs. control; Fig 5A). The proliferation rate of IPAH-PASMCs was dramatically augmented in the presence of 10 μM R568 after 48 h culture. As the enhancing effect of R568 at 48 h was most prominent (Fig 5Ba), we evaluated the dose-response relationship of PASMC proliferation at 48 h. The R568-induced increase in cell number occurred in a concentration-dependent manner (185±15% at 10 μM, n = 11, p<0.01 vs. control; Fig 5Bb), with an EC_50_ value of 0.33 μM and a Hill coefficient of 2.10 (n = 7~11). Similarly to normal PASMCs, the proliferation of CTEPH-PASMCs was slightly facilitated during the exposure to R568 for 48 h (117±4% at 10 μM, n = 5, p = 0.049 vs. control; Fig 5C).

### Effects of CaSR modulators on PASMC proliferation by BrdU incorporation assay

**Fig 6:**
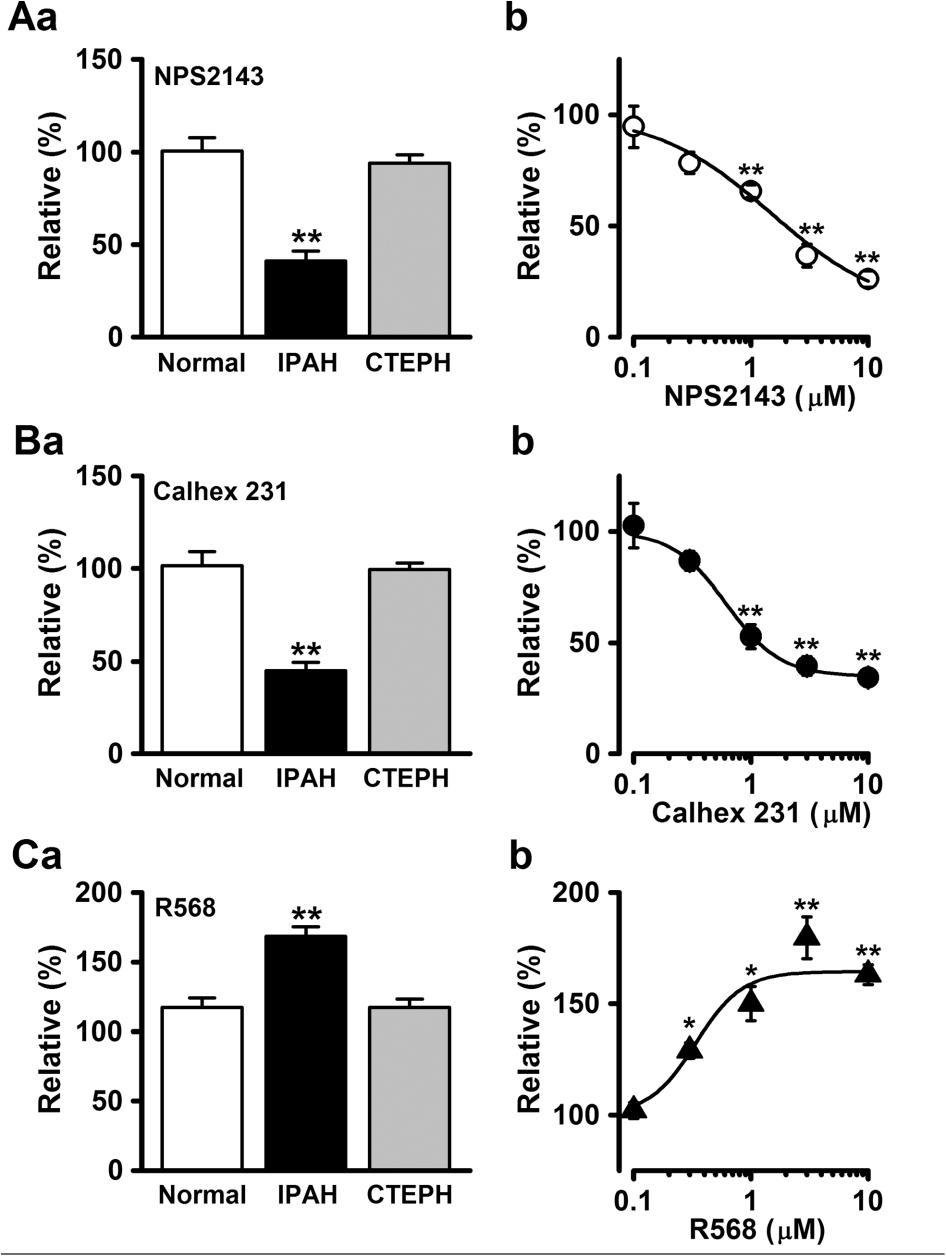
Effect of CaSR modulators on cell proliferation using BrdU incorporation assay. The proliferation rates of PASMCs from normal subjects, IPAH patients, and CTEPH patients were analyzed using quantitative colorimetric assay based on the BrdU incorporation method for cell proliferation. The effects of NPS2143 (**A**), Calhex 231 (**B**), and R568 (**C**) on cell proliferation were examined in normal, IPAH, and CTEPH PASMCs. Summarized data (**a**) show the effect of CaSR modulators at a concentration of 3 μM on cell proliferation at 48 (for R568) or 72 (for NPS2143 and Calhex 231) h in normal, IPAH, and CTEPH PASMCs. Dose-response curve (**b**) of cell proliferation for NPS2143, Calhex 231, and R568 in IPAH-PASMCs. The absorbance was normalized by the value in the absence of drug (as 100%). The IC_50_ values of NPS2143 and Calhex 231 for the proliferation of IPAH-PASMCs were 1.48 and 0.62 μM, respectively. The EC_50_ value of R568 was 0.34 μM. Data were obtained from 7~14 experiments. *p<0.05 or **p<0.01 vs. control (100%).

To confirm our results obtained from the MTT assay, another quantitative colorimetric assay based on the BrdU incorporation method for cell proliferation was carried out in normal, IPAH, and CTEPH PASMCs. The excessive proliferation of IPAH-PASMCs was markedly reduced by the treatment with 3 μM NPS2143 for 72 h culture (41±5%, n = 8, p<0.01 vs. control (100%); Fig 6Aa). The inhibitory effect by NPS2143 on cell proliferation occurred in a concentration-dependent manner (26±4% at 10 μM, n = 8, p<0.01; Fig 6Ab). The IC_50_ value of NPS2143 was 1.48 μM, and the Hill coefficient was 0.89 (n = 8). The application of 3 μM Calhex 231 also inhibited the excessive cell proliferation in IPAH-PASMCs (45±4% at 72 h, n = 9, p<0.01; Fig 6Ba), and the inhibition occurred concentration-dependently (34±4% at 10 μM, n = 8, p<0.01; Fig 6Bb), with an IC_50_ value of 0.62 μM and a Hill coefficient of 1.89 (n = 8). The cell proliferation in IPAH-PASMCs was enhanced by 3 μM R568 (169±7% at 48 h, n = 7, p<0.01; Fig 6Ca). The augmented cell proliferation occurred in a concentration-dependent manner (163±4% at 10 μM, n = 8, p<0.01; Fig 6Cb) with an EC_50_ value of 0.34 μM and a Hill coefficient of 2.19 (n = 8). In contrast, the proliferation of normal and CTEPH PASMCs was not significantly changed by 3 μM NPS2143 (101±7%, n = 9 and 94±4%, n = 7, respectively, p>0.05), 3 μM Calhex 231 (102±8%, n = 9 and 99±4%, n = 7, respectively, p>0.05), or 3 μM R568 (117±7%, n = 10, p = 0.085 and 117±6%, n = 14, p = 0.127, respectively).

## Discussion

Our previous report showed that the excessive proliferation rate of IPAH-PASMCs was attenuated by siRNA knockdown of CaSR [19]; however, pharmacological modulators are required for the development of drug therapy for PAH. In this investigation, we have shown that calcilytics (NPS2143 and Calhex 231) inhibit the excessive proliferation of PASMCs from IPAH patients, whereas a calcimimetic (R568) enhances the proliferation of PASMCs from IPAH patients as well as from normal subjects and CTEPH patients. Similar results were obtained from two different experiments for cell proliferation based on MTT test and BrdU incorporation assay. These results indicate that the activity of CaSR contributes to the PASMC proliferation.

[Ca^2+^]_cyt_ plays an important role in regulation of the proliferation and migration of PASMCs. An increase in [Ca^2+^]_cyt_ in PASMCs is an important stimulus for PASMC proliferation and pulmonary vascular remodeling under physiological and pathological conditions [6, 7, 10]. We recently found that CaSR is upregulated in lung tissues and PASMCs isolated from IPAH patients, as well as in animals with experimental pulmonary hypertension [19, 26, 27]. This upregulated CaSR plays a pivotal role in the increased resting [Ca^2+^]_cyt_ and augmented Ca^2+^ influx, as well as the enhanced cell proliferation in IPAH-PASMCs. Importantly, the intraperitoneal injection of the calcilytic NPS2143 prevented the development of pulmonary hypertension and right ventricular hypertrophy in monocrotaline-induced pulmonary hypertensive rats and hypoxia-induced pulmonary hypertensive mice. Functionally upregulated CaSR in PASMCs may be involved in a novel pathogenic mechanism underlying excessive pulmonary vascular remodeling in IPAH patients. In this study, we have direct evidence *in vitro* that calcilytics specifically inhibit the excessive proliferation of IPAH-PASMCs. Interestingly, calcilytics did not affect the cell growth of normal PASMCs. Pharmacological blockade of the upregulated CaSR with calcilytics may be a novel therapeutic approach for PAH patients who do not respond to conventional drug therapy. On the other hand, a calcimimetic enhanced the proliferation of IPAH-PASMCs. Even for normal and CTEPH PASMCs, cell growth was slightly but significantly increased by a calcimimetic. This result is in agreement with our previous observation that CaSR is expressed at low levels in the plasma membrane of normal PASMCs [19].

CaSR belongs to the G-protein-coupled receptor subfamily C [20, 21, 28]. The activation of CaSR coupled to Gq proteins stimulates phospholipase C that hydrolyzes phosphatidylinositol 4,5-bisphosphate to inositol 1,4,5-trisphosphate (IP_3_) and diacylglycerol. IP_3_ induces Ca^2+^ release from the sarcoplasmic reticulum mediated by IP_3_ receptors. In vascular smooth muscle cells, the activation of CaSR increases [Ca^2+^]_cyt_ and thereafter induces vasoconstriction under physiological conditions [29–31]. Therefore, CaSR is thought to be involved in the regulation of myogenic tone, peripheral vascular resistance, and arterial blood pressure [32–34]. Alternatively, CaSR also regulates multiple signal pathways such as mitogen-activated protein kinase (MAPK) cascades: MAPK kinase (MEK), extracellular signal-regulated kinases (ERK1/2), and c-Jun N-terminal kinase (JNK). Recently obtained evidence suggests that CaSR in vascular myocytes contributes to cell proliferation and apoptosis through MAPK and phospholipase C cascades [35–38]. It is likely that [Ca^2+^]_cyt_ increase mediated by CaSR activation triggers MAPK cascades to accelerate PASMC proliferation under physiological and pathological conditions. Therefore, the blockade of CaSR by calcilytics caused attenuation of excessive cell proliferation in IPAH-PASMCs. Related to the decrease in cell viability by the treatment with calcilytics, these cells apparently exhibited altered morphological features, leading to outcomes such as cell damage and death. Calcilytics may cause apoptosis and necrosis followed by morphological damage involving cell death. However, further experiments are necessary to elucidate the mechanism underlying cell proliferation and death via the CaSR pathway.

In pharmacological research associated with CaSR, NPS2143, Calhex 231, and R568 are widely used as CaSR modulators [39, 40]. A prototypical calcimimetic, R568 (2-chloro-N-[(1R)-1-(3-methoxyphenyl)ethyl]benzenepropanamine) (Fig 1B), is a phenylalkylamine compound structurally derived from fendiline, a blocker of voltage-dependent Ca^2+^ channels [41]. On the other hand, the first calcilytic, NPS2143 (2-chloro-6-[(2R)-3-[[1,1-dimethyl-2-(2-naphthalenyl)ethyl]amino-2-hydroxypropoxy]benzonitrile), was not obtained by direct derivatization based on phenylalkylamine calcimimetics, but by optimization of a positive compound in high-throughput screening [42] (Fig 1A). Later on, Calhex 231 (4-chloro-N-[(1S,2S)-2-[[(1R)-1-(1-naphthalenyl)ethyl]amino]cyclohexyl]benzamide) was developed as a calcilytic structurally unrelated to NPS 2143 by another high-throughput screening [43]. In this study, three structurally-unrelated CaSR modulators influenced the proliferation of IPAH-PASMCs; namely, inhibition or enhancement by calcilytics or calcimimetic, respectively. Furthermore, the IC_50_ and EC_50_ values of these modulators on the proliferation of IPAH-PASMCs were close to the effective concentration ranges of CaSR in recombinant HEK293 cells [40–43] and vascular smooth muscle cells [19, 27, 44, 45]. To our knowledge, there are few reports about adverse effects of these compounds. Taken together, our data strongly support our conclusion that these compounds interact with CaSR to respond in PASMCs.

On the basis of our understanding of the pathological mechanisms of PAH, drug therapy for PAH has progressed in recent years via the development of several specific drugs that offer an effective alternative to voltage-dependent Ca^2+^ channel blockers (calcium blockers), such as nifedipine and diltiazem [2, 28]. Currently, three therapeutic classes are widely used for the treatment of PAH: endothelin receptor antagonists (bosentan and ambrisentan), phosphodiesterase type 5 inhibitors (sildenafil and tadalafil), and prostacyclins (also known as prostaglandin I_2_) (epoprostenol, treprostinil, and iloprost). Moreover, numerous potential candidates have either been submitted for approval or are in development. Despite recent major therapeutic advances, current treatments of PAH do not achieve a cure of life-threatening disease. In this study, we have demonstrated that the PASMC proliferation is modulated by the activity of CaSR, which is upregulated in IPAH patients, and that this is a novel pathogenic mechanism underlying the augmented PASMC proliferation in IPAH patients. These results also provide an important pathway for developing therapeutic interventions for PAH. Calcilytics are potential drug candidates for the treatment of osteoporosis and other bone metabolism diseases [39, 40]. Therefore, the accumulation of clinical data about calcilytics may be useful for clinical trials and the application of calcilytics as therapeutic drugs for PAH.

Our previous reports showed that a calcilytic inhibited the development of pulmonary hypertension in monocrotaline-induced pulmonary hypersensitive rats and hypoxia-induced pulmonary hypertensive mice [19, 26]. In this study, we clearly demonstrate that calcilytics attenuate the excessive proliferation of PASMCs from IPAH patients, whereas calcilytics do not affect cell survival in PASMCs from normal subjects. Taken these findings together, calcilytics are a potential candidate for therapeutic drugs for PAH patients.
